# Pharmacogenetic inhibition of TrkB signaling in adult mice attenuates mechanical hypersensitivity and improves locomotor function after spinal cord injury

**DOI:** 10.3389/fncel.2022.987236

**Published:** 2022-09-26

**Authors:** Karmarcha K. Martin, Donald J. Noble, Shangrila Parvin, Kyeongran Jang, Sandra M. Garraway

**Affiliations:** Department of Cell Biology, Emory University School of Medicine, Atlanta, GA, United States

**Keywords:** BDNF, mechanical hypersensitivity, plasticity, spinal cord injury, TrkB, pERK

## Abstract

Brain-derived neurotrophic factor (BDNF) signals through tropomyosin receptor kinase B (TrkB), to exert various types of plasticity. The exact involvement of BDNF and TrkB in neuropathic pain states after spinal cord injury (SCI) remains unresolved. This study utilized transgenic TrkBF616 mice to examine the effect of pharmacogenetic inhibition of TrkB signaling, induced by treatment with 1NM-PP1 (1NMP) in drinking water for 5 days, on formalin-induced inflammatory pain, pain hypersensitivity, and locomotor dysfunction after thoracic spinal contusion. We also examined TrkB, ERK1/2, and pERK1/2 expression in the lumbar spinal cord and trunk skin. The results showed that formalin-induced pain responses were robustly attenuated in 1NMP-treated mice. Weekly assessment of tactile sensitivity with the von Frey test showed that treatment with 1NMP immediately after SCI blocked the development of mechanical hypersensitivity up to 4 weeks post-SCI. Contrastingly, when treatment started 2 weeks after SCI, 1NMP reversibly and partially attenuated hind-paw hypersensitivity. Locomotor scores were significantly improved in the early-treated 1NMP mice compared to late-treated or vehicle-treated SCI mice. 1NMP treatment attenuated SCI-induced increases in TrkB and pERK1/2 levels in the lumbar cord but failed to exert similar effects in the trunk skin. These results suggest that early onset TrkB signaling after SCI contributes to maladaptive plasticity that leads to spinal pain hypersensitivity and impaired locomotor function.

## Introduction

Chronic neuropathic pain is a frequent consequence of spinal cord injury (SCI; Siddall and Loeser, 2001; Felix et al., 2007). Yet, limited progress has been made in the development of effective treatments, mainly because little is known about the underlying neural mechanisms. Complex interactions between the primary insult and various secondary processes, including the engagement of numerous cellular mediators, have been implicated in the processes that promote pain (see reviews by Hulsebosch, 2002; Hulsebosch et al., 2009). Among these cellular mediators is the neurotrophin, brain-derived neurotrophic factor (BDNF), which signals through its high affinity tropomyosin receptor kinase B (TrkB), which can be expressed as two isoforms—full-length (TrkB145) or truncated TrkB (TrkB95). Earlier studies showed that BDNF is upregulated in small sensory neurons after peripheral inflammation induced by nerve growth factor or intra-plantar complete Freund’s adjuvant (Apfel et al., 1996; Cho et al., 1997; Michael et al., 1997). BDNF can function as a neuromodulator and has been shown to modulate synaptic transmission from C fibers to dorsal horn neurons, thereby contributing to central sensitization and inflammatory pain (Kerr et al., 1999; Thompson et al., 1999).

Building on the study by Kerr et al. (1999), we undertook electrophysiological studies to evaluate the cellular and synaptic effects of BDNF and showed that BDNF facilitates afferent-evoked synaptic responses and NMDA-induced inward currents in lamina II neurons in acute lumbar spinal cord slices (Garraway et al., 2003). However, the facilitatory effects of BDNF were eliminated following transection or contusion of the spinal cord (Garraway et al., 2005; Garraway and Mendell, 2007), an effect that was linked to an injury-induced increase in GABA_A_-mediated inhibition. Despite our results, several studies have shown that elevated BDNF levels promote adaptive plasticity (Gomez-Pinilla et al., 2001, 2007; Baker-Herman et al., 2004; Baumbauer et al., 2009; Huie et al., 2012) and functional recovery (Hutchinson et al., 2004; Ying et al., 2005; Boyce et al., 2012) after injury. Altogether, these studies show that BDNF-TrkB signaling exerts different types of plasticity after SCI (see Garraway and Huie, 2016).

As it relates to pain, we recently reported that BDNF and TrkB levels are decreased in the spinal cord after SCI, at or near the lesion epicenter (Garraway et al., 2011; Strickland et al., 2014), at the same time-point when mechanical hypersensitivity is observed (Garraway et al., 2014). Because increases (rather than decreases) in BDNF and TrkB are seen in conditions of inflammatory and nerve injury-induced pain, these observations suggest that spinal BDNF minimally contributes to pain after SCI. However, it is possible that BDNF contributes to pain through non-central actions, as BDNF-TrkB signaling in the periphery is critical to various functions and plasticity. For example, whereas BDNF in primary afferents does not play a significant role in acute pain, it is necessary for some chronic inflammatory and neuropathic pain states (Sikandar et al., 2018). Also, BDNF released from epithelial cells in the skin plays a role in normal mechanosensation by engaging TrkB receptors in the hair follicles of cutaneous afferents (Li et al., 2011; Rutlin et al., 2014; also see Carroll et al., 1998).

This study proposes that BDNF-TrkB signaling underlies neuropathic pain, presumably involving non-spinal mechanisms, and undertakes a first-step investigation into the specific contribution TrkB signaling makes to pain hypersensitivity after SCI. To accomplish this, we examined the effect of systemic pharmacogenetic inhibition of TrkB on evoked pain responses after a thoracic (T)10 SCI on adult mice. Comparative studies were also undertaken following intra-plantar formalin injection. We used a transgenic mouse (TrkBF616) that expresses a mutated TrkB (Chen et al., 2005), and administered 1NM-PP1, a small molecule, cell permeable kinase inhibitor selectively binds to the mutated receptor and reversibly blocks TrkB signaling (e.g., Chen et al., 2005; Johnson et al., 2008; Greising et al., 2017).

Normally, when BDNF engages its full-length TrkB receptor, it activates mitogen-activated protein kinase/extracellular signal-regulated kinase (MAPK/ERK), phospholipase C-γ (PLC-γ), and phosphatidylinositol-3 kinase (PI3-K) intracellular signaling cascades (Kaplan and Miller, 2000; Chao, 2003; Huang and Reichardt, 2003; Blum and Konnerth, 2005; Wang et al., 2009). Activation of these kinases, including ERK, leads to post-translational modifications such as phosphorylation and translation. Phosphorylated ERK1/2 (pERK1/2) is a downstream kinase that mediates the cellular effects of ERK and is implicated in inflammatory and neuropathic pain (Zhuang et al., 2005; Crown et al., 2006; Xu et al., 2008). Importantly, Chen et al. (2005) showed that in the absence of the inhibitor, TrkB signaling is maintained in the TrkBF616 mice, therefore making it a useful model to explore the role TrkB signaling plays in pain hypersensitivity. Overall, this study examined the effect of systemic blockade of TrkB signaling on inflammatory and neuropathic pain responses. We also assessed the effect of TrkB inhibition on expression levels of pERK and total ERK, as well as TrkB receptor isoforms.

## Methods

### Subjects

Experiments were performed in TrkBF616 (JAX # 022363) mice which were bred in our animal colony, and wild-type C57BL/6 (JAX # 000664) mice of both sexes. Mice were approximately 3–4 months old at the time of formalin administration or surgery and weighed 20–22 g (females) and 24–26 g (males). They were housed in standard cages in a vivarium on a 12:12-h light-dark cycle with all behavioral testing performed during the light period. Animals were fed standard rodent diets *ad libitum*. Experimental procedures were approved by the Animal Care and Use Committee of Emory University, and conformed to national standards for the care and use of experimental animals and the American Physiological Society’s “Guiding Principles in the Care and Use of Animals”.

### Drug administration and pharmacological verification

TrkBF616 mice were treated with 1-(1, 1-dimethylethyl)-3-(1-naphthalenylmethyl)-1H-pyrazolo[3,4-d]pyrimidin-4-amine (1NM-PP1 or 1NMP; Cayman Chemical # 13330). 1NMP is a cell permeable kinase inhibitor that blocks TrkB receptor autophosphorylation and hence TrkB signaling. 1NMP was dissolved in Dimethyl Sulfoxide (DMSO) and then added to drinking water at a final concentration of 5 μM. For vehicle (Veh)-treated control, an equal volume of DMSO was added to drinking water (dilution ratio, 1:10,000). Mice were allowed to drink 1NMP or Veh-treated water for 5 days, consuming approximately 30–40 milliliters (ml). Comparable procedures for pharmacogenetic inhibition of TrkB signaling by 1NMP administration have been previously reported (e.g., Wang et al., 2009; Rantamäki et al., 2011; Akhter et al., 2019).

To demonstrate the effectiveness and specificity of 1NMP inhibition, electrophysiological and pharmacological studies were also undertaken in acutely dissociated dorsal root ganglia (DRG) neurons obtained from vehicle- and 1NMP-treated TrkBF616 mice. Whole-cell patch clamp recording of small-diameter cultured neurons was performed. The neurons were superfused with the BDNF agonist, 7,8-dihydroxyflavone (7,8-DHF, 100 μm) in artificial Cerebrospinal Fluid for 1 min. Detailed methodologies and results of these studies are provided as Supplementary Material.

### Effect of TrkB inhibition on formalin-induced inflammatory pain

BDNF or TrkB signaling has been implicated in inflammatory pain (e.g., Lin et al., 2011). As a prelude to our SCI-related studies, we first tested whether pharmacogenetic blockade of TrkB, in adult mice blocks inflammatory pain as was similarly done by Wang et al. (2009). This first experiment that was undertaken in TrkBF616 and wild-type mice was an important approach to validate the use of the mutant mice in the subsequent behavioral studies. TrkBF616 mice in this experiment were treated with the inflammatory agent formalin on the last day of 1NMP (F616-1NMP) or vehicle (F616-Veh) treatment in drinking water. A group of wild-type mice was also included in this experiment for comparison. For further validation of the specificity of 1NMP-induced inhibition of TrkB in the mutants, a cohort of wild-type mice was treated with 1NMP for 5 days prior to formalin administration (Wild-type-1NMP).

#### Formalin test

Mice were acclimated to the behavioral suite and test chamber for several days prior to experimentation. The test chamber was an acrylic animal chamber (IITC Life Science #433) placed on a wire-mesh tabletop. On the test day (day 5 of 1NMP or vehicle treatment), they were placed in the testing chamber for 30 min. At time zero and while under mild hand-held restraint, 20 μl of a 5% formalin solution (5% formaldehyde in 0.9% saline) was administered subcutaneously to the dorsal surface of one hind-paw, in a right-left counterbalanced manner. Mice were immediately returned to the testing chamber for 60 min. Video recording for the pre- and post-formalin periods was done for subsequent analysis of nociceptive responses of the injected hind-paw. Two trained individuals, who were blinded to the treatment, manually and independently counted the nociceptive responses, characterized as lifts and flinches, in 1-min bins. Twenty-four hours after formalin, the animals were euthanized, and the lumbar (L2–5) spinal cord sections were removed for Western blot analyses as described below.

### Effect of TrkB inhibition on pain and locomotor function after SCI

#### Animal and surgical procedure

TrkBF616 mice were used in this experiment. The mice were deeply anesthetized with isoflurane (5%, gas; lowered to 2%–3% once stable anesthesia was achieved). Under sterile conditions, a skin incision and dorsal laminectomy exposed the underlying spinal cord at T10. For midline contusion injuries, mice received a 70 kdyne, zero dwell time, impact onto the dorsal surface of the spinal cord with an Infinite Horizon Impactor (Precision Systems and Instrumentation, Fairfax Station, VA), as we previously described (Parvin et al., 2021). Care was taken to ensure that the laminectomy or impact did not damage the dorsal roots, and on-target bilateral bruising of the dorsal spinal cord was verified by examination under a dissecting microscope. The overlying muscle and skin were sutured, and the wound area was treated with triple antibiotic ointment (bacitracin-neomycin-polymyxin B) topically. Sham control mice underwent the same surgical procedure but without receiving an impact to the spinal cord. Mice were given meloxicam [5 mg/kg, subcutaneously (SC)] and lactated Ringer’s solution [0.5 ml, intraperitoneally (IP)] immediately after surgery, and left to recover on a heated pad. They were also administered 0.9% sterile saline daily (0.5 ml) for the first 48 h after surgery to maintain hydration. Subsequent administration of saline was given only as needed. Mice received the antibiotic Baytril (2.5 mg/kg, SC) immediately after surgery and daily each morning up to 7 days post-operation (dpo) to minimize the risk of the urinary tract or bladder infection in SCI animals. Experimenters manually expressed mouse bladders twice daily for the duration of experiments. Mice were assessed for impairment of locomotor function at 1 dpo using the Basso Mouse Scale (BMS; Basso et al., 2006), to ensure the effectiveness of the injury. SCI mice were only included in the study if they recorded BMS scores of 0 or 1 at 1 dpo.

#### Drug administration

SCI and Sham mice were treated with 1NMP or vehicle in drinking water as described above. Thus, these experiments consisted of the following four experimental groups: Sham-Veh, Sham-1NMP, SCI-Veh, and SCI-1NMP. To assess the effect TrkB inhibition has on the onset of pain after SCI, SCI and sham control mice were treated with 1NMP or vehicle from day of surgery (day 0) to day 5 (Early Treatment group). A second group of SCI and Sham mice were treated from 16–21 days after SCI, to examine the effect of TrkB inhibition on the maintenance of hind-paw hypersensitivity (Late Treatment group).

#### Assessment of mechanical hypersensitivity (allodynia)

At baseline (before surgery) and weekly (7, 14, 21 and 28 dpo), mice underwent the von Frey test of mechanical sensitivity. All mice were acclimated to the behavioral suite and testing apparatuses for at least 3 days prior to surgery. On the day of testing, they were acclimatized to the testing apparatus for 20 min. Mechanical assessment was undertaken according to the established up-down method (Chaplan et al., 1994). Briefly, calibrated von Frey hairs (NC12775-99, North Coast Medical, Inc., Morgan Hill, CA, USA) starting with filament evaluator size 3.22 (target force 0.16 g) were administered from below a metal mesh platform to test each animal’s sensitivity to mechanical stimulation of the hind-paw. Right and left paw withdrawal thresholds were averaged to determine overall mechanical sensitivity. A reduction in von Frey withdrawal threshold values from their baseline levels indicated enhanced mechanical hypersensitivity.

#### Conditioned Place Aversion (CPA) test

To provide a validated assessment of at-level, affective pain after SCI, we used a modified two-chamber CPA paradigm similar to those described in previous studies (Hummel et al., 2008; Yang et al., 2014; Bagdas et al., 2016; Refsgaard et al., 2016; Wu et al., 2017). The custom-built CPA apparatus consisted of black (dark) and white (light) chambers separated by a small partition. Each CPA box contained a narrow horizontal window permitting entry of a small brush (Camel hair #4, Ted Pella, Inc., Redding, CA, USA) for manual stimulation. CPA testing was run from ~30 to 34 dpo, reflecting the post-SCI mechanical hypersensitivity at chronic time points. For the pre-conditioning test (~30 dpo), mice were placed in the light chamber and given free access to the entire apparatus for a 20-min test period (with 10 additional minutes for habituation) to determine initial time spent in each chamber. They then received daily 30-min conditioning sessions over the course of 3 days (~31–33 dpo). On each day, animals were administered brush stimulation in their preferred chambers (once/minute for 15 min) delivered over a distance of ~3 cm across the trunk in the caudal-to-rostral direction at a speed of ~1 cm/s, while they were partially restrained in acrylic cylinders (allowing some movement but not 180 degree changes in orientation; Rodent Restrainer, IITC Life Science, Woodland Hills, CA; Noble et al., 2019). In the control, non-preferred chambers, mice received pseudo “stimulation” whereby the brush was swept across the abdominal area but made no direct contact, while animals were under similar partial physical restraint (again once/minute for 15 min). We used a counterbalanced design so that approximately half of the mice were exposed to the control chamber first and half to the stimulation chamber. One day after completion of the conditioning phase (~34 dpo), each mouse was again allowed free access to explore both chambers for a 20-min post-conditioning test (again excluding 10 min habituation) with no stimuli present. Video recordings were collected throughout testing periods, and the percentage of time spent in the non-stimulated chamber before and after stimulation were taken to indicate relative place aversion. Immediately after the day 5 assessment (34 dpo), the mice were euthanized, and lumbar spinal cord excised and used for Western blot as described below.

#### Assessment of locomotor function

A cohort of mice was used to assess the effect of early (days 0–5) vs. late (days 16–21) administration of 1NMP or vehicle on recovery of locomotor function. BMS locomotor assessments were done at 1, 3, 7, 14, 21, and 28 dpo in SCI and Sham subjects. Assessments were done by two individuals who were blinded to treatment administered.

### Effect of TrkB inhibition on TrkB, pERK1/2, and ERK1/2 expression

At the end of the behavioral assessments, the mice were deeply anesthetized with isoflurane and 1 cm of lumbar spinal cord encompassing L2–L5 was rapidly removed. In 22 mice (18 SCI and four Sham subjects), 0.5 cm^2^ of truncal skin was also removed. Protein was extracted using RIPA lysis buffer, and quantified using the bicinchoninic acid protein assay. Western blot was undertaken as we previously described (Garraway et al., 2011). Briefly, 30 μg of total protein were subjected to SDS-PAGE using 12% Tris–HCl precast gels (Thermo Scientific, Rockford, IL, USA) and then transferred to PVDF membrane (Millipore, Bedford, MA). For quantification of non-phosphorylated proteins (TrkB, ERK and β-tubulin), the blots were blocked in 5% blotting grade milk (Bio-Rad Laboratories, Hercules, CA) in Tris-buffered saline Tween-20 (TBST), whereas phospho-ERK blots were blocked in 5% bovine serum albumin in TBST. The blots were incubated overnight at 4°C in primary antibodies generated in rabbit for the full-length (145 kDa) and truncated (95 kDa) forms of the TrkB receptor (1:500; #07-225-Upstate Cell Signaling, Lake Placid, NY), ERK1/2 (1:2,000; #06-182-Millipore, Temecula, CA) and pERK1/2 (1:500; #07-467-Millipore, Temecula, CA), and β-tubulin (1:1,000; #05-661-Upstate Cell Signaling, Lake Placid, NY) which was generated in mouse. The following day, blots were washed in TBST, then incubated in HRP-conjugated goat anti-rabbit or anti-mouse secondary antibodies (1:5,000; #31460 or 31430, Thermo Fisher Scientific, Waltham, MA), and developed with standard enhanced chemiluminescence and imaged with Azure Biosystems c400 Western blot Imaging System. Ratios of the integrated densitometry of each protein of interest to the loading control (β-tubulin) were calculated with AlphaView Software by ProteinSimple, normalized to wild-type or Sham-Veh controls and averaged for animals within each group.

### Statistical analyses

All statistical measures and analyses were undertaken with GraphPad Prism v9 (GraphPad Software, La Jolla, CA), with significance set at *p* < 0.05. Although it was impossible to blind experimenters to SCI vs. Sham groups, experimenters performing behavioral tests and subsequent statistical analyses were blinded to treatment (1NMP vs. vehicle treatment). Comparison between groups was accomplished using 2-way repeated measures (RM) ANOVA with days post-operation as the within-subjects factor and injury condition/drug treatment as the between-subjects factor. Three-way ANOVA was not performed, since in order to maintain sufficient statistical power, this would have required assignment of additional mice to subgroups that were not essential to test our hypotheses. For example, SCI-1NMP mice were split into early and late treatment groups to test the effects of TrkB inhibition on locomotor function, and wild-type mice were compared with F616–1NMP and F616-Veh mice to test the effects of TrkB inhibition on formalin-induced inflammatory pain. Two-way ANOVA with “group” (injury condition combined with treatment) as the between-subjects factor was the most practical analytical choice to avoid unnecessary comparisons while minimizing use of experimental animals. Western blot data were analyzed using 1-way ANOVA. For all statistical analyses, *post-hoc* tests corrected for multiple comparisons were performed in the case of significant results. In all cases, the choice of multiple comparisons test was selected according to GraphPad Prism (taking appropriateness for the particular dataset into account). Paired or unpaired *t*-tests were occasionally performed as planned comparisons, as indicated in the text. In text and figures, all data are presented as Mean ± SEM. In the figures, * and ^#^ (*p* < 0.05), ** and ^##^ (*p* < 0.01), *** and ^###^ (*p* < 0.001) and **** (*p* < 0.0001) indicate significant effects.

## Results

### Pharmacological assessment of DRG neurons

As detailed in Supplementary Material, eight TrkBF616 mice were used for electrophysiological and pharmacological examination of TrkB inhibition by 1NMP. Bath administration of 7, 8-DHF induced an inward current in acutely dissociated DRG neurons. As shown in Supplementary Figure 1, neurons obtained from 1NMP-treated mice (*n* = 4) showed a significant attenuation of agonist-induced inward current compared to vehicle-treated mice (*n* = 4; *p* < 0.0001, *t test*). These results indicate that administration of 1NMP in drinking water to TrkBF616 mice effectively blocks TrkB signaling.

### Effect of TrkB inhibition on formalin-induced inflammatory pain

#### Formalin-induced behavioral response

**Figure 1:**
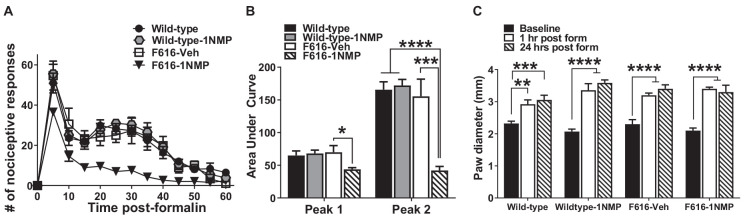
Inhibition of TrkB attenuates formalin-induced inflammatory pain responses. **(A)** Intraplantar administration of formalin induced a biphasic nociceptive response characterized as phase 1 (0–10 min) and phase 2 (11–60 min) in three (Wild-type, Wild-type-1NMP and F616-Veh) of the four experimental groups of mice. There were no differences in cumulative number of responses between Wild-type, Wild-type-1NMP, and vehicle-treated F616 mice except at 60 min, where Wild-type was different to F616-Veh (*p* = 0.02). The number of responses in 1NMP-treated F616 mice was significantly reduced compared to wild-type and F616-Veh mice at binned times ranging from 15 to 50 min and from wild-type-1NMP at 5–55 min (p values ranging from *p* < 0.05 to *p* < 0.0001; 2-way ANOVA with Tukey’s multiple comparisons tests). **(B)** Quantitative measurement of the formalin response (area under the curve; AUC) revealed that F616–1NMP mice had a significantly reduced phase 1 response compared to F616-Veh treated mice only (*p* < 0.05), while their phase 2 response was robustly decreased compared to both wild-type groups (*p* < 0.0001) and F616-Veh group (*p* < 0.001). **(C)** Formalin induced significant paw edema in all four treatment groups, which was evident at both 1 and 24 h post-formalin (*p < 0.05; **p < 0.01; ***p < 0.001; and ****p < 0.0001).

Twenty-nine male and female mice were treated with formalin [Wild-type (*n* = 8), Wild-type-1NMP (*n* = 7), F616–1NMP (*n* = 7) and F616-Veh (*n* = 7)]. Preliminary analyses indicated no sex difference, and hence results for both males and females are combined. Formalin produces a characteristic biphasic response with phase 1 characterized as 0–10 min and phase 2 as 11–60 min post-injection. Between phase 1 and 2 is an interphase of decreased activity (Coderre et al., 1993). The cumulative nociceptive response(s) per 5 min bin was plotted vs. the time after formalin injection in Figure 1A. As shown, both wild-type groups and F616-Veh treated mice displayed the normal biphasic response. A 2-way RM ANOVA revealed a significant group difference in number of responses among the four groups (*F*_(3, 25)_ = 15, *p* < 0.0001), and a significant group × time interaction (*F*_(36, 300)_ = 2, *p* = 0.0001) that was controlled by F616–1NMP mice showing fewer nociceptive responses to formalin injection compared to Wild-type, Wild-type-1NMP and F616-Veh mice during phase 2. Multiple comparisons tests on the cumulative number of responses in each 5 min bin revealed significant group differences from 5 to 55 min after formalin (p values ranging from *p* < 0.0001 to *p* = 0.04; 2-way ANOVA). In all cases, F616 mice treated with 1NMP had a reduction in nociceptive responses post-formalin. At 60 min after formalin, there were no differences in the number of nociceptive responses between F616 mice receiving 1NMP and the other three groups. Measurement of the area under the curve (AUC; Figure 1B) showed similar results. A 1-way ANOVA revealed significant group differences in the phase 1 (*F*_(3, 25)_ = 3, *p* = 0.038) and phase 2 (*F*_(3, 25)_ = 17, *p* < 0.0001) responses. As shown in Figure 1B, a much more robust group effect was seen in the phase 2 response, which was substantially reduced in F616–1NMP mice compared to the other groups. Importantly, wild-type mice treated with 1NMP had nociceptive responses that were indistinguishable from their untreated counterparts. These results confirmed a role for TrkB signaling in inflammatory pain and established that administration of 1NMP for 5 days effectively blocks TrkB signaling in TrkBF616 mice.

#### Formalin induced paw edema

Formalin is known to induce a pronounced local edema. To assess whether TrkB inhibition also blocks the peripheral swelling, paw diameters were measured using a calibrated caliper, which was applied midpoint across the dorsal to plantar surface of the injected hind-paws. Measurements were made before formalin (Baseline) and at 1 h and 24 h after injection. As shown in Figure 1C, formalin produced robust peripheral swelling in the affected hind-paws at 1 and 24 h after treatment in all four animal groups [Wild-type (*F*_(2, 24)_ = 10.5, *p* = 0.0005), Wild-type-1NMP (*F*_(2, 18)_ = 36.2, *p* < 0.0001), F616-Veh (*F*_(2, 21)_ = 22.8, *p* < 0.0001) and F616–1NMP (*F*_(2, 18)_ = 26.8, *p* < 0.0001), 1-way ANOVA with *post-hoc* tests comparing baseline with 1 and 24 h]. This observation suggests that the diminished nociceptive response following formalin injection in F616–1NMP mice is not due to 1NMP blocking the peripheral edema.

#### Formalin-induced changes in spinal TrkB, pERK, and ERK protein levels

**Figure 2:**
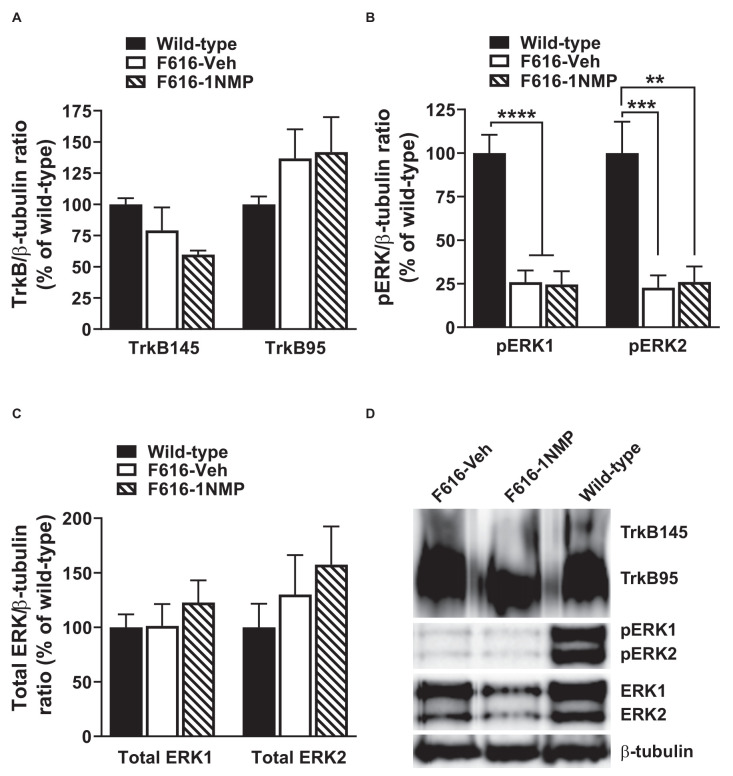
Spinal expression of TrkB, pERK, and ERK in formalin inflammatory pain. **(A,C)** There were no differences in the spinal protein expression of TrkB and ERK isoforms, respectively, among the three groups of mice 24 h after formalin injection. **(B)** The levels of both pERK1 and 2 were significantly decreased in the F616 mice compared to Wild-type, although 1NMP treatment had no effect on pERK1/2 expression. **(D)** Representative Western blot images are shown for TrkB, pERK, and ERK expression in the lumbar spinal cord (**p < 0.01; ***p < 0.001; and ****p < 0.0001).

Formalin-induced pain is associated with the engagement of kinases and subsequent phosphorylation of membrane proteins (e.g., Garraway et al., 2009). Here, we assessed the effect of formalin and TrkB inhibition on the expression of TrkB95, TrkB145, pERK1/2 and total ERK1/2 in the lumbar spinal cord, 24 h after formalin. Because the behavioral results revealed no differences in the wild-type groups, one wild-type group was used in these assessments. As shown in Figure 2A, 1-way ANOVA revealed no differences in the expression of either of the TrkB isoforms among the three treatment groups. This outcome suggests that there is minimal impact of TrkB inhibition on TrkB expression during inflammatory pain. Surprisingly, pERK levels were significantly reduced in both F616 treatment groups, compared to the wild-type group. This was true for pERK1 (*F*_(2, 14)_ = 26.4, *p* < 0.0001) and pERK2 (*F*_(2, 14)_ = 14.1, *p* = 0.0004). It is unlikely that decreased levels of pERK are inherent to the TrkBF616 mice given the previous report by Chen et al. (2005). There were no differences in the pERKs expression levels between the F616-Veh and F616–1NMP groups (Figure 2B), despite the differences observed in the formalin-induced nociceptive responses. Unlike pERK, there were no differences in the protein expression levels of ERK1 and 2 among the wild-type and F616 groups (Figure 2C). These results indicate different expression patterns of total ERK and activated ERK levels in the spinal cord of wild-type and TrkBF616 during formalin-induced inflammatory pain. Examples of representative western blot images for TrkB, pERK1/2, ERK1/2, and β-tubulin are shown in Figure 2D.

### Effect of TrkB inhibition on SCI-induced behavioral responses

#### Mechanical hypersensitivity after SCI

This experiment explored the effect of TrkB inhibition on hind-paw mechanical sensitivity up to 4 weeks after SCI. Studies were undertaken in both male and female mice. However, as preliminary analyses revealed no sex differences, the results are combined.

##### Early treatment group

**Figure 3:**
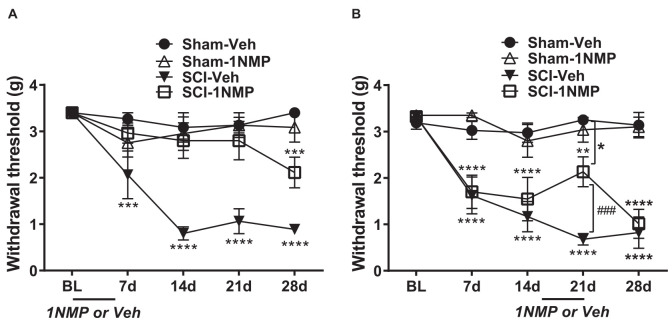
TrkB inhibition attenuates hind-paw hypersensitivity after SCI. **(A)** Early administration of 1NMP (days 0–5) after SCI (open squares) blocked the development of mechanical hypersensitivity seen in SCI-Veh mice (filled triangles) which developed significant mechanical hypersensitivity at all timepoints (7–28 dpo) after SCI compared to their pre-surgery baselines (***p < 0.001, and ****p < 0.0001). Only at 28 dpo did SCI-1NMP mice show a decrease in withdrawal threshold compared to baseline (***p < 0.001). However, even then, their average withdrawal threshold was significantly greater than that of the vehicle-treated counterparts at 28 dpo (*p* < 0.01, not indicated on graph). There were no differences in the withdrawal thresholds of either Sham group across treatment days. **(B)** Both SCI “late treatment” groups developed mechanical hypersensitivity over time compared to baseline withdrawal thresholds (**p < 0.01 and ****p < 0.0001). Treatment with 1NMP at 16–21 days after SCI (open squares), reversibly attenuated mechanical hypersensitivity at 21 dpo [withdrawal thresholds: significantly higher than SCI-Veh (^###^*p* < 0.001); although significantly lower than the Sham-Veh group (*p = 0.05)]. At 28 dpo, mechanical thresholds of SCI-1NMP mice had returned to those seen at 7 and 14 dpo, and were indistinguishable from SCI-Veh treated mice (filled triangles). SCI-Veh mice displayed hind-paw hypersensitivity at all time points, compared to their pre-surgery baselines. Sham mice did not show differences in withdrawal threshold across timepoints.

Mice in this group received 1NMP-treated water from day of surgery to day 5 (days 0–5). A 2-way RM ANOVA revealed a main effect of group/treatment (*F*_(3, 24)_ = 20.0, *p* < 0.0001) and time (*F*_(4, 96)_ = 11.7, *p* < 0.0001), and a significant group × time interaction (*F*_(12, 96)_ = 4.6, *p* < 0.0001). As shown in Figure 3A and revealed through Dunnett’s multiple comparisons tests, neither of the sham groups (*n* = 6 each) developed mechanical hypersensitivity at any time point post-surgery compared to baseline, whereas SCI-Veh mice (*n* = 8) developed mechanical hypersensitivity at 7 dpo (*p* = 0.0003), which was maintained at all later time points tested (*p* < 0.0001 at 14, 21, and 28 dpo). In contrast, SCI-1NMP mice (*n* = 8) did not develop mechanical hypersensitivity until much later at 28 dpo (Dunnett’s multiple comparisons test: *p* = 0.0005). The reductions in hind-paw withdrawal thresholds of SCI-Veh mice (filled triangles) at post-SCI timepoints compared to baseline became substantial over time [7 dpo (2.1 ± 0.5 g), 14 dpo (0.8 ± 0.1 g), 21 dpo (1.1 ± 0.3 g), and 28 dpo (0.9 ± 0.1 g) vs. baseline (3.4 ± 0 g)]. In contrast, only at 28 dpo did 1NMP-treated SCI mice (open squares) exhibit sensitivity thresholds (2.1 ± 0.3 g), that approached that seen early on (7 dpo) in SCI-Veh mice. Nonetheless, even at 28 dpo, *post-hoc* testing indicated a significant difference in response threshold between the SCI-Veh (0.9 ± 0.1 g) and SCI-1NMP (2.1 ± 0.3 g) treated groups (*p* = 0.006). *Post-hoc* comparisons also revealed that there were no differences in paw sensitivities between the two sham groups, between the two 1NMP groups, or between Sham-Veh and SCI-1NMP groups, whereas SCI-Veh differed significantly from Sham-Veh, Sham-1NMP, and SCI-1NMP groups throughout the testing period (*p* < 0.0001 in all cases). Overall, these results show an acute blockade of pain sensitivity by TrkB inhibition, thereby delaying the onset of hind-paw hypersensitivity after SCI.

##### Late treatment group

Mice in this group were treated with 1NMP in drinking water on days 16–21 after surgery. The effect of 1NMP or vehicle treatment on established mechanical responses after SCI is shown in Figure 3B. A 2-way RM ANOVA revealed a main effect of group/treatment (*F*_(3, 30)_ = 22.5, *p* < 0.0001) and time (*F*_(4, 120)_ = 19.3, *p* < 0.0001), and a significant group × time interaction (*F*_(12, 120)_ = 5.8, *p* < 0.0001). Dunnett’s multiple comparisons tests confirmed that neither sham group developed mechanical hypersensitivity at any time point post-surgery, whereas SCI-Veh and SCI-1NMP mice displayed increased sensitivity at all time points compared to baseline (SCI-Veh: *p* < 0.0001 at 7, 14, 21, and 28 dpo; SCI-1NMP: *p* = 0.0011 at 21 dpo; *p* < 0.0001 at 7, 14, and 28 dpo; *n* = 9 each group). While the SCI-Veh (filled triangles) and SCI-1NMP (open squares) groups showed a similar effect, in SCI-1NMP mice there was a slight reversal of mechanical sensitivity at 21 dpo (2.1 + 0.3 g) toward the baseline sensitivity (3.3 + 0.1 g). Interestingly, the response was not completely reversed, as sensitivity at 21 dpo was not different from that observed at 7 or 14 dpo. Furthermore, *post-hoc* tests comparing SCI group sensitivities at each time point revealed that there were no differences between the SCI-Veh and SCI-1NMP groups on days 7, 14, and 28 (*p* > 0.05); however, at 21 dpo, mechanical thresholds in SCI-Veh mice (0.7 ± 0.1) were significantly reduced compared to SCI-1NMP mice (2.1 ± 0.3; *p* < 0.001). At 21 days, SCI-1NMP thresholds were significantly lower than those of the Sham-Veh group (*p* < 0.05) but not the Sham-1NMP group. *Post-hoc* comparisons also revealed that there were no differences in paw sensitivities between the two sham groups (*n* = 8 each group) at any of the time points. These results suggest that 1NMP effectively attenuated already-established pain hypersensitivity, although the response returned within a week.

#### Conditioned Place Aversion (CPA) responses after SCI

**Figure 4:**
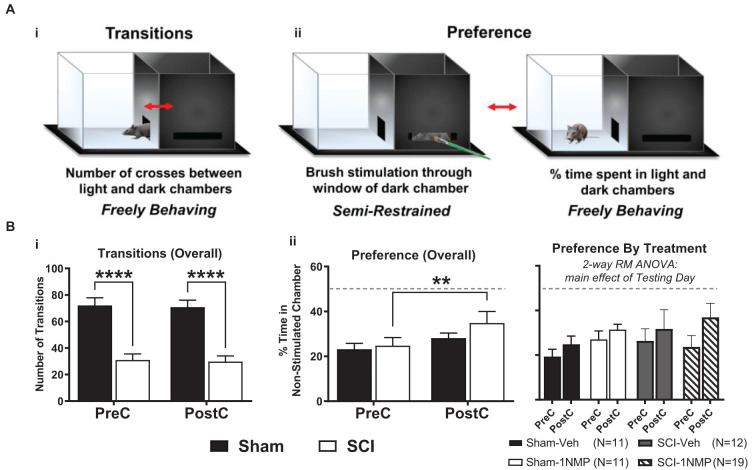
TrkB inhibition does not block conditioned place aversion (CPA). **(A)** The dual chamber (“light and dark”) CPA apparatus with a mouse shown inside, visually depicting how: **(i)** side-to-side transitions are quantified, and **(ii)** animal preferences are determined by monitoring time spent in each chamber during periods of free exploration, before and after brush stimulation. **(B) (i)** SCI mice had fewer transitions before and after stimulation thereby revealing continued locomotor impairment several weeks after injury (****p < 0.0001 SCI vs. Sham). **(ii, left)** SCI mice receiving mechanical brush stimulation while partially restrained in the dark chamber developed a preference for the light chamber post-stimulation (**p < 0.005 pre vs. post). **(ii, right)** Results broken down by treatment (vehicle or 1NMP) for all SCI and Sham mice, illustrating that 1NMP treatment did not alleviate affective pain behavior. PreC, pre-conditioning; PostC, post-conditioning. The dashed horizontal line in **(B)** indicates 50% time spent in each chamber (no preference).

The brush stimulation CPA cohort consisted of 53 total TrkBF616 mice (*n* = 22 sham and *n* = 31 SCI, comprising subjects from all four experimental groups). No differences emerged between early and late treatment cohorts; therefore, the results are combined for presentation (Figure 4). A light-dark box CPA paradigm was used to assess afferent-mediated pain aversion (contextual aversion to directionally selective mechanical brush stimulation). Chamber transitions and time spent in each chamber (preference) were monitored as depicted in Figure 4A. For side-to-side transitions between chambers (Figure 4Bi), there was a significant main effect of group in a 2-way RM ANOVA (SCI vs. Sham; *F*_(1, 51)_ = 39.4, *p* < 0.0001), with SCI mice performing significantly fewer transitions at each time point (*p* < 0.0001 for both pre- and post-conditioning), but there were no differences from pre-conditioning to post-conditioning in Sham (*t*_(21)_ = 0.3, *p* > 0.05, paired *t*-test) or SCI (*t*_(30)_ = 0.4, *p* > 0.05, paired *t*-test) mice. For chamber preference, a 2-way RM ANOVA revealed a main effect of testing day (pre vs. post; *F*_(1, 51)_ = 11.6, *p* < 0.005), and *post-hoc* comparisons using Šídák’s multiple comparisons tests showed that SCI mice underwent a significant change in chamber preference from pre- to post-conditioning (*p* < 0.005), amounting to a 10.2% increase in preference for the non-stimulated chamber (Figure 4Bii). Sham mice did not undergo a similar change in preference (*p* > 0.05), and no difference in preference was evident between SCI and Sham mice for pre-conditioning (*t*_(51)_ = 0.3, *p* > 0.05, unpaired *t*-test) or post-conditioning (*t*_(51)_ = 1.1, *p* > 0.05, unpaired *t*-test). The change in preference we observed in SCI mice following stimulation was not different between mice receiving vehicle and 1NMP, as indicated by 2-way RM ANOVA: while there was a main effect of testing day (pre vs. post; *F*_(1, 49)_ = 10.0, *p* < 0.005), there was no effect of treatment group (SCI-1NMP, SCI-vehicle, Sham-1NMP, or Sham-vehicle; *F*_(3, 49)_ = 0.5, *p* > 0.05). Further analyzing SCI-1NMP mice by time of treatment using unpaired *t*-tests revealed no differences between early and late treatment in chamber preference during pre-conditioning (*t*_(17)_ = 0.02, *p* > 0.05) or post-conditioning (*t*_(17)_ = 0.5, *p* > 0.05). These results support the emergence of at-level supraspinal pain following SCI, an effect that appears to be resistant to TrkB inhibition.

#### Locomotor function after SCI

**Figure 5:**
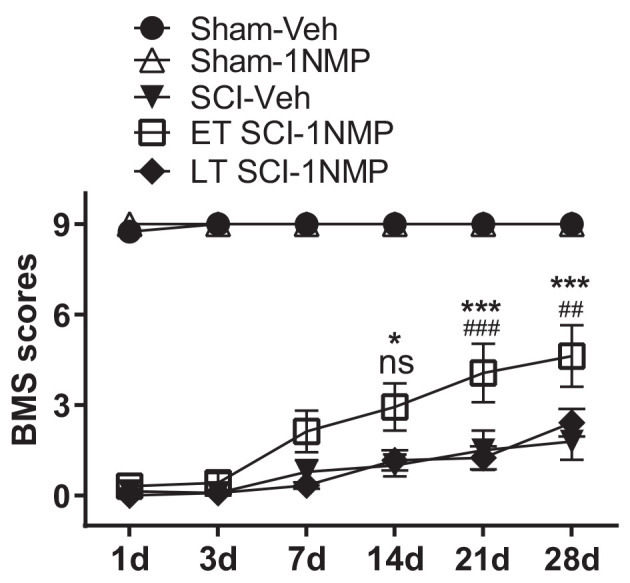
Early inhibition of TrkB signaling with 1NMP improves locomotor recovery. SCI mice treated with 1NMP at 0–5 dpo had significantly improved BMS scores at 7, 14, 21 and 28 days compared to day 1 score (*p* < 0.0001; not indicated). Importantly, early 1NMP treated mice (ET SCI-1NMP) had higher BMS scores than late treated mice (LT SCI-1NMP, 16–21 dpo) at 21 dpo (^###^*p* < 0.001) and 28 dpo (^##^*p* < 0.01), and SCI-Veh mice at 14 dpo (*p < 0.05), 21 and 28 dpo (***p < 0.001 in both cases), ns indicates no significant difference between early and late treated SCI mice. SCI mice in all three groups had impaired locomotor function compared to Sham groups.

A distinct cohort of mice were used for locomotor assessment as follows: Sham-Veh (*n* = 4); Sham-1NMP (*n* = 7); SCI-Veh (*n* = 8); SCI-1NMP, early treatment (ET SCI-1NMP; *n* = 8) and SCI-1NMP, late treatment (LT SCI-1NMP; *n* = 6). To increase statistical power, animals in the Sham-Veh, Sham-1NMP and SCI-Veh groups included both early and late vehicle or 1NMP treatment. As expected, there were no differences in locomotor responses based on time of treatment in these three groups. BMS locomotor scores were assessed at 1, 3, 7, 14, 21, and 28 dpo. Due to several randomly distributed missing data points, mixed-effects analysis with Tukey’s multiple comparison tests was undertaken to investigate changes in locomotor scores among the five groups over time. There was a significant effect among treatment groups (*F*_(4, 27)_ = 106, *p* < 0.0001), with all three SCI groups recording significantly lower BMS scores compared to the Sham groups over all six test days. Comparing the SCI groups, ET SCI-1NMP group (open squares) had significantly improved locomotor scores compared to SCI-Veh mice (closed triangles) at 14 (*p < 0.05), 21 and 28 dpo (***p < 0.001) and LT SCI-1NMP mice (closed diamonds) at 21 and 28 dpo (^###^*p* < 0.001 and ^##^*p* < 0.01, respectively; Figure 5). Consistent with improved locomotor function, ET SCI-1NMP mice had higher BMS scores at 7, 14, 21, and 28 dpo compared to their day 1 scores (*p* < 0.0001 in all cases; not shown on graph). In contrast, SCI-Veh and LT SCI-1NMP mice began to recover locomotion only at 21 dpo (*p* = 0.009 and *p* = 0.03, respectively) with more appreciably recovery at 28 dpo (*p* = 0.0009 and *p* < 0.0001, respectively). Overall, these results show an improvement in hind-limb locomotor recovery following early inhibition of TrkB signaling after SCI.

### Effect of TrkB inhibition on TrkB, pERK, and ERK protein expression in lumbar spinal cord and trunk skin after SCI

**Figure 6:**
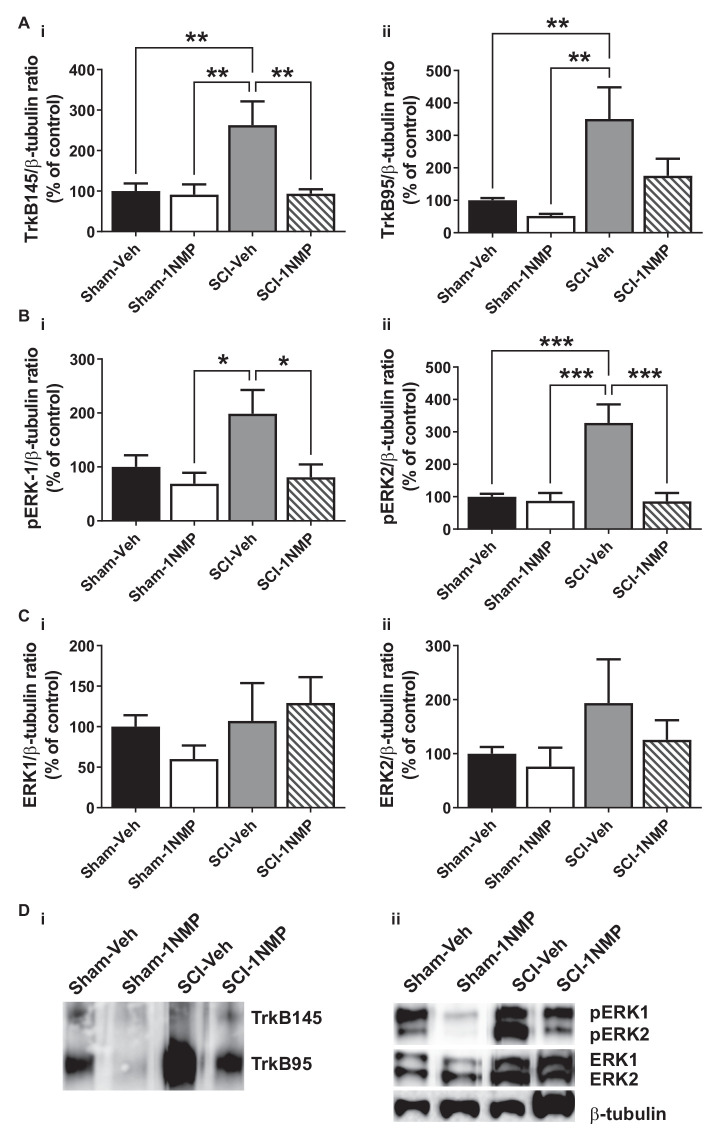
Early inhibition of TrkB signaling reduces spinal TrkB and pERK expression. **(A) (i,ii)** SCI increased both full length and truncated TrkB expression and, **(B) (i,ii)** pERK1 and pERK2 levels in the lumbar spinal cord at 34 dpo. Early treatment with 1NMP (day 0–5) blocked these increases. For both TrkB and pERK proteins, the SCI-1NMP group did not differ in expression levels from the Sham-Veh or Sham-1NMP groups. **(C) (i,ii)** Total ERK expression levels were unchanged among the four experimental groups (*p < 0.05, **p < 0.01, and ***p < 0.001; 1-way ANOVA, *n* = 6, each group). **(D)** Representative images of **(i)** TrkB, and **(ii)** pERK1/2, ERK1/2, and β-tubulin (loading control) Western blot are shown.

**Figure 7:**
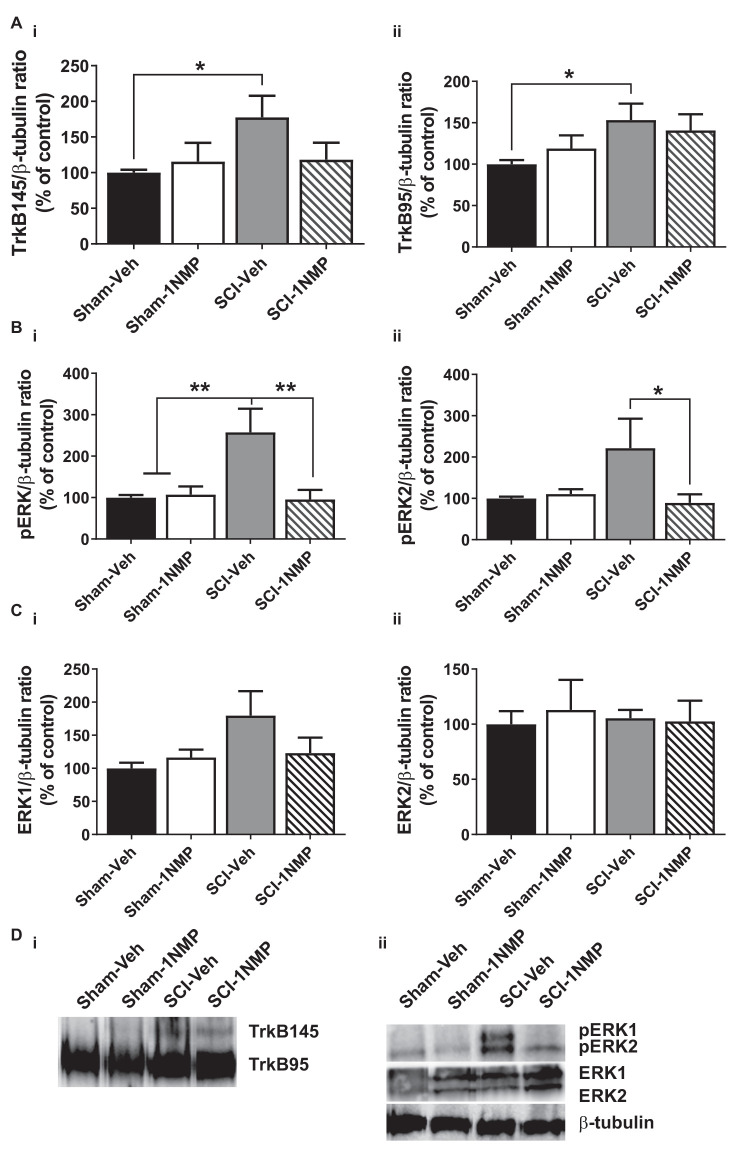
Late inhibition of TrkB signaling modestly reduces spinal TrkB and pERK expression. **(A) (i,ii)** TrkB expression was significantly increased in the lumbar spinal cord of SCI-Veh mice compared to Sham-Veh subjects only at 34 dpo (*p < 0.05, *unpaired t test*). **(B) (i,ii)** Both pERK1 and pERK2 levels were reduced following 1NMP treatment compared to vehicle treatment 16–21 days after SCI (*p < 0.05, and **p < 0.01, 1-way ANOVA). However, only pERK1 levels were significantly increased in SCI-Veh mice in comparison to Sham subjects (**p < 0.01, 1-way ANOVA). **(C) (i,ii)** Total ERK expression levels were unchanged among the four experimental groups (*n* = 7, each group). **(D)** Representative images of **(i)** TrkB, and **(ii)** pERK1/2, ERK1/2, and β-tubulin (loading control) Western blot are shown.

**Figure 8:**
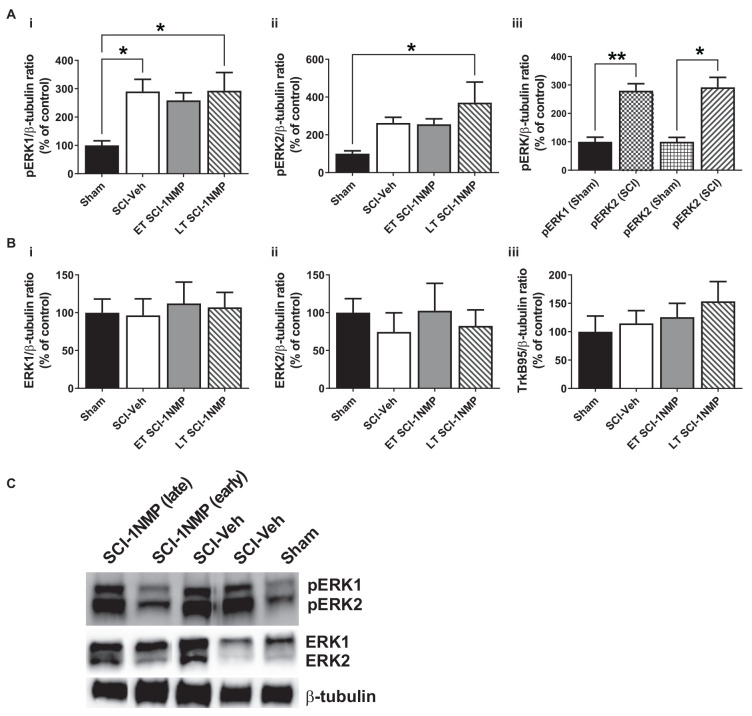
SCI increases pERK expression in the adjacent trunk skin. **(A) (i,ii)** SCI increased the expression of pERK1 and 2 in the adjacent trunk skin compared to sham procedures, although neither early nor late treatment with 1NMP was able to prevent the increases (*p < 0.05, 1-way ANOVA).** (iii)** Overall, SCI produced a robust increase in trunk pERK1 and pERK2 levels compared to sham procedure (*p < 0.05, **p < 0.01, *unpaired t test*). **(B)** Neither **(i,ii)** ERK1/2 nor **(iii)** TrkB95 levels were changed in the trunk skin among the experimental groups. **(C)** Representative images of pERK1/2, ERK1/2, and β-tubulin (loading control) Western blot are shown.

Engagement of the TrkB145 receptor activates several kinases including ERK (see review by Huang and Reichardt, 2003). Activated ERK (pERK) is implicated in spinal pain processing underlying inflammatory and neuropathic pain (Zhuang et al., 2005; Crown et al., 2006; Xu et al., 2008). Truncated TrkB has been shown to exert inhibitory effects on BDNF signaling (Eide et al., 1996). To better understand potential peripheral and central mechanisms, Western blot analyses were performed to assess whether pharmacogenetic inhibition of TrkB differentially modulates the protein expression levels of the TrkB receptor isoforms TrkB145 (full-length), TrkB95 (truncated), pERK1/2 and total ERK1/2 in the lumbar spinal cord (Figures 6 and 7) and skin (Figure 8). These cellular quantifications were done at 34 dpo regardless of when 1NMP or vehicle treatments were done.

In mice receiving 1NMP- or vehicle-treated water early (0–5 days after SCI), 1-way ANOVA comparing the four groups showed that there was a significant group effect in both TrkB145 (*F*_(3, 17)_ = 7.1, *p* = 0.003) and TrkB95 (*F*_(3, 17)_ = 7.8, *p* = 0.002; Figures 6Ai,ii). Tukey’s multiple comparisons tests showed that the TrkB isoforms were significantly elevated in the spinal cord of SCI-Veh mice compared to the Sham groups. Also, TrkB145 was attenuated in the SCI-1NMP group compared to SCI-Veh mice. Similarly, there were significant group effects for pERK1 (*F*_(3, 18)_ = 4.2, *p* = 0.02) and pERK2 (*F*_(3, 18)_ = 13.2, *p* < 0.0001; Figures 6Bi,ii). Both pERK isoforms were increased in SCI-Veh mice but reduced in the SCI-1NMP group. There were no differences in the total ERK1 and ERK2 expression among the groups (Figures 6Ci,ii).

In the late treatment groups (16–21 days after SCI; Figure 7), 1-way ANOVAs did not reveal significant differences in TrkB95 and TrkB145 expressions between the four treatment groups. However, when direct comparisons between the Sham and SCI vehicle-treated groups were made, there was a significant increase in both TrkB isoforms in SCI-Veh subjects compared to Sham-Veh (*p* < 0.05, *unpaired t tests*; Figures 7Ai,ii). Significant group differences were observed in both pERK1 (*F*_(3, 22)_ = 6.9, *p* = 0.002) and pERK2 (*F*_(3, 22)_ = 3.5, *p* = 0.03) expression. Most notably, 1NMP treatment attenuated pERK1/2 levels compared to vehicle treatment after SCI (Figures 7Bi,ii). ERK1 and ERK2 levels were unchanged by any of the treatments (Figures 7Ci,ii). Altogether, it appears that early 1NMP treatment caused a more robust attenuation of SCI-induced increases in TrkB isoforms, whereas pERK levels were decreased by 1NMP treatment irrespective of when 1NMP was provided.

The protein expression of TrkB, pERK, and ERK in the trunk skin was also measured by Western blot in a cohort of subjects [Sham (*n* = 4), SCI-Veh (*n* = 6), ET SCI-1NMP (*n* = 7) and LT SCI-1NMP (*n* = 5)]. As shown in Figures 8Ai,ii, there was a significant group effect for the pERK1 (*F*_(3, 17)_ = 3.8, *p* = 0.030) but not pERK2 (*F*_(3, 17)_ = 3.1, *p* = 0.053) expression, although pERK2 levels in LT SCI-1NMP mice were significantly increased compared to Sham only (*p* < 0.05). There were no differences among the three SCI groups, hence 1NMP treatment, irrespective of time, did not attenuate pERK1/2 expression levels in the skin. However, when all three SCI groups were aggregated and compared to the Sham control group, unpaired t tests revealed significant increases in pERK1 (*p* < 0.01) and pERK2 (*p* < 0.05) in the trunk skin of SCI mice compared to Sham control subjects (Figure 8Aiii). There were no differences among the four groups in ERK1, ERK2 and TrkB95 expression (Figures 8Bi,iii, respectively). Note that we did not observe TrkB145 in the skin tissue as seen in the spinal cord.

## Discussion

The specific role that BDNF-TrkB signaling plays in the development or maintenance of neuropathic pain states after SCI is unknown. In this study, we explored a novel approach to determine if TrkB signaling contributes to pain hypersensitivity after SCI. Using TrkBF616 mice that permit reversible pharmacogenetic inhibition of TrkB, we showed that systemic inhibition of TrkB with 1NMP treatment significantly attenuated neuropathic pain responses after SCI, as well as reduced nociceptive responses in formalin-induced inflammatory pain. We also observed that TrkB inhibition immediately after SCI improved locomotor recovery. At the cellular level, 1NMP treatment prevented the increases in spinal pERK1/2 expression otherwise seen after SCI (SCI-vehicle treated).

It is not surprising that TrkB inhibition significantly reduced formalin-induced pain responses, as BDNF-TrkB signaling is implicated in inflammatory pain (Kerr et al., 1999; Groth and Aanonsen, 2002; Matayoshi et al., 2005; Zhao et al., 2006). While Wang et al. (2009) did not observe an attenuation of formalin responses in TrkBF616 mice (unlike our current result), they showed that mechanical hypersensitivity post-formalin and CFA-induced pain, were reduced in mice treated with 1NMP for 2 days. Because the second phase of the formalin response occurs by central mechanisms (Sawynok and Liu, 2003), we expected TrkB inhibition to alter spinal pERK expression levels, which we and others have shown to be critical to inflammatory pain (Ji et al., 2002; Adwanikar et al., 2004; Xu et al., 2008). However, pERK levels were unchanged by 1NMP and furthermore, we observed an unexpected decrease in pERK in TrkBF616 mice compared to wild-type mice. This cellular change was not reflected in the behavioral outcome, as there were no differences in nociceptive responses between wild-type and F616-Veh mice, although both exhibited a greater response than F616–1NMP mice. It is possible that at the time when the cellular assessment was performed, the formalin response had waned. Previously, we observed elevated phosphorylated NMDAR1 levels approximately 45 min after formalin injection (Garraway et al., 2009), which suggests that kinase activation might occur shortly after formalin administration. Nonetheless, the current observations show that TrkB signaling is critical to the development of inflammatory pain, although its contributions to the underlying central cellular mechanisms remain to be elucidated. Furthermore, it justifies the TrkB616 mice as a valid model for assessing behavioral and cellular outcomes following TrkB inhibition.

The major objective of this study was to investigate the effect of TrkB inhibition on pain responses after SCI. Hence, a significant outcome is that TrkB inhibition effectively attenuated the onset and maintenance of mechanical hypersensitivity after SCI. The demonstration that pharmacogenetic inhibition of TrkB immediately after SCI significantly delayed the onset of pain hypersensitivity to 4 weeks is profound, in that it establishes a critical role for TrkB signaling in the development of mechanical hypersensitivity after SCI. It also suggests that early interventions, presumably before the peak of numerous secondary effects, could mitigate devastating outcomes after SCI. Although TrkB inhibition effectively reduced mechanical hypersensitivity, CPA responses (regardless of the time of 1NMP treatment) were unaffected. SCI mice showed an increased preference for the non-stimulated chamber compared to Sham control subjects, but there was no difference among the two SCI treatment groups. This result was unexpected as it suggests that TrkB signaling has minimal effect on supraspinal pain measures. However, this seems improbable as BDNF signaling is widely shown in the anterior cingulate cortex, an area implicated in conditioned place preference and pain responses (Zhang et al., 2016, 2018; Miao et al., 2021). The lack of effect of 1NMP on CPA responses could be due to the duration of TrkB inhibition. It is possible that at ~5 weeks after SCI, when CPA is undertaken, the inhibitory effect of 1NMP has waned in these mice. Equally likely is the possibility that TrkB contributes to pain hypersensitivity primarily during the acute post injury time. This is highly probable given that TrkB inhibition immediately after SCI blocked the onset of mechanical hypersensitivity up to at least 4 weeks, unlike treatment with 1NMP at 2–3 weeks post injury which only partially and reversibly attenuated mechanical hypersensitivity. Similarly, early TrkB inhibition caused a more robust attenuation of spinal TrkB levels than treatment at a later time-point. One final possibility is that behavioral variability and the smaller magnitude of group differences (SCI vs. Sham) in CPA compared to the von Frey test effectively obscured any additional effects of 1NMP treatment on the supraspinal pain assessment. However, additional studies need to be undertaken to fully reconcile the differential outcomes of TrkB signaling on supraspinal vs. spinal-mediated pain responses.

That inhibition of TrkB immediately after SCI improved the recovery of locomotion was an unexpected outcome. Prior studies have consistently shown treatments that increase BDNF levels and TrkB signaling in the spinal cord enhance adaptive plasticity (Huie et al., 2012) and improve locomotor recovery (Jakeman et al., 1998; Liu et al., 1999; Hutchinson et al., 2004; Boyce et al., 2012) after SCI. Nonetheless, our results suggest there is a critical window of vulnerability for TrkB signaling after SCI. Although it is possible that the improvement in locomotion following early 1NMP treatment could be related to a reduction in pain responses, this however cannot fully account for these results, as no short-term improvement in locomotion was seen at 21–28 days after treatment in the late 1NMP-treated SCI mice. Thus, it appears that TrkB engages distinct locomotor and pain networks within the spinal cord immediately after SCI. Unlike most of the prior studies where BDNF-TrkB signaling was targeted at least 1 week after SCI, here, TrkB blockade commenced immediately after SCI. Consequently, we show for the first time that early onset TrkB signaling has the potential to produce maladaptive plasticity, leading to pain hypersensitivity and compromised locomotor recovery.

Supporting these findings, Matyas et al. (2017) previously showed that a major truncated TrkB isoform, TrkB.T1, is upregulated in the spinal cord at 7 days after SCI in different cell types, although it declines by 8 weeks. They also showed that TrkB.T1 signaling in astrocytes underlies neuropathic pain and locomotor deficits after SCI (also see Cao et al., 2020). It appears that TrkB signaling, albeit *via* its truncated receptor, promotes devastating outcomes after SCI, possibly by inhibiting otherwise adaptive effects of BDNF. Truncated TrkB isoforms, including TrkB.T1, have been shown to function as dominant negative receptors, exerting inhibitory effects on BDNF signaling (Eide et al., 1996). Therefore, overexpression of TrkB95 after SCI could serve to limit any beneficial plasticity exerted by BDNF signaling through its full-length receptor. Here, we observed that both TrkB95 and 145 protein levels were strongly upregulated in the lumbar spinal cord after SCI. Interestingly, although early treatment with 1NMP attenuated both isoforms, a greater reduction was seen with the full-length isoform (Figure 6A). Although this study did not directly distinguish the specific effects of TrkB95 vs. TrkB145 isoforms, we can postulate that early engagement of TrkB signaling after SCI impacts both sensory and motor functions.

Overall, the current study introduces a novel view into the role of systemic TrkB signaling in pain and locomotion after SCI. We previously reported independent studies that BDNF-TrkB signaling at or near the lesioned spinal cord might not contribute to pain after SCI. Specifically, BDNF fails to facilitate afferent-evoked synaptic responses in lamina II neurons after SCI (Garraway et al., 2005; Garraway and Mendell, 2007), and both BDNF and TrkB levels are decreased in the injured spinal cord even when mechanical hypersensitivity is observed (Garraway et al., 2014). In fact, numerous additional studies have shown that BDNF and TrkB are decreased in the lesioned spinal cord (King et al., 2000; Liebl et al., 2001; Hajebrahimi et al., 2008; Garraway et al., 2011; Strickland et al., 2014), although TrkB expression could be increased in spinal segments distal to the injury, as we report here. As previously mentioned, BDNF promotes adaptive plasticity after SCI. However, the previous belief that TrkB signaling broadly promotes adaptive plasticity after SCI is being challenged by our current results. By employing well-characterized models of peripheral inflammation and SCI, we provide evidence that systemic TrkB signaling contributes to pain hypersensitivity, and impedes locomotor recovery after SCI.

Because 1NMP is cell permeable and crosses the blood-brain barrier, the exact site of action is unknown although some general conclusions can be made. TrkB inhibition at early time points abrogated SCI-induced increases in pERK and TrkB expression in the lumbar spinal cord. TrkB signaling activates several kinases, including ERK, and BDNF increases TrkB signaling by autocrine actions. Because central sensitization results in events such as protein synthesis and receptor trafficking (Latremoliere and Woolf, 2009), TrkB inhibition can have broad effects on central cellular mechanisms that promote pain. We also examined whether TrkB inhibition similarly mitigates peripheral plasticity. We observed significant increases in pERK1/2 expression in the trunk skin after SCI, overlapping with the area that received truncal stimulation during the CPA paradigm. Increased expression of pERK1/2 in the skin might play a critical role in promoting at-level allodynia. Elevated pERK in peripheral nerve terminals innervating the plantar skin was observed following noxious stimulation and concurrent with capsaicin-induced thermal hyperalgesia (Dai et al., 2002). Although we did not characterize the specific cell types that express pERK, peripheral plasticity including hyperexcitability of nociceptors is presumed to underlie this phenotype.

Despite the robust increase in pERK1/2 expression in the skin, the levels were not attenuated by 1NMP treatment, as was seen with spinal pERK1/2 levels. Furthermore, TrkB inhibition did not attenuate peripheral edema following formalin. These findings strongly support the notion that TrkB signaling that drives pain hypersensitivity and locomotor deficits resides in the spinal cord, although peripheral involvement cannot be entirely ruled out. It was recently shown that BDNF in hairy skin is required for normal functioning of a sub-population of cutaneous afferents known as the Aδ-Low-Threshold Mechanoreceptor (LTMRs; Li et al., 2011; Rutlin et al., 2014). Aδ-LTMRs express TrkB and transduce directional touch. BDNF-TrkB signaling is required for the normal directionality function of the Aδ-LTMRs. BDNF is also expressed in a population of myelinated primary afferents, although according to Dembo et al. (2018), its expression in these primary afferents makes very limited contributions to pain or itch. However, a second study targeting primary afferent-derived BDNF showed that deletion of BDNF from peripheral sensory neurons resulted in deficits in inflammatory and neuropathic pain responses (Sikandar et al., 2018). Hence, BDNF and TrkB dysfunction in the periphery could contribute to pain after SCI, even though this has not yet been definitively shown.

This study investigated the effect of TrkB signaling on pain hypersensitivity by employing selective pharmacogenetic inhibition of TrkB in mouse models of inflammatory and SCI-induced neuropathic pain. The results strongly suggest that TrkB signaling produces maladaptive plasticity that contributes to mechanical hypersensitivity, and locomotor dysfunction after SCI. Moreover, engagement of these maladaptive pathways immediately after SCI appears to have a more pronounced effect on outcomes. Although these results substantiate a role for TrkB signaling in neurological outcomes after SCI, we are not able to ascertain the specific contributions of central vs. peripheral TrkB signaling mechanisms. Similarly, whether early blockade of TrkB signaling remains effective in the more chronic stages of injury is unknown. Finally, some concern is warranted regarding the specificity of 1NMP treatment in these mice, given the potential widespread actions of small molecule kinase inhibitors (Bishop et al., 2000; Bain et al., 2007). However, this concern was allayed by our data showing that 1NMP-treated wild-type mice showed formalin-induced nociceptive responses that were identical to untreated mice, and that treatment with 1NMP substantially reduced TrkB-agonist induced currents in sensory neurons. Notwithstanding these limitations, the study clearly demonstrates that TrkB signaling, which is usually perceived as a facilitator of adaptive plasticity after SCI, can induce detrimental actions, presumably by engaging cellular kinase pathways in the immediate aftermath of SCI. Furthermore, it highlights a potentially novel neural mechanism underlying pain after SCI, that is one involving TrkB signaling. The outcomes of this study support the notion that early intervention targeting TrkB signaling could produce beneficial outcomes after SCI. Importantly, the key finding from this research emphasizes the need for further investigation into the role of BDNF-TrkB signaling in chronic neuropathic pain after SCI.

## Data Availability Statement

The original contributions presented in the study are included in the article/Supplementary material, further inquiries can be directed to the corresponding author.

## Ethics Statement

The animal study was reviewed and approved by Animal Care and Use Committee of Emory University.

## Author Contributions

KM performed SCI, and undertook all SCI-related behavioral tests, assisted with writing and editing the manuscript. DN performed conditioned place tests and data analyses and assisted with writing the manuscript. SP assisted with SCI-related behavioral studies, performed Western blots and edited the manuscript. SG led the scientific oversight of the project, assisted with experiments, performed data analyses, and wrote the manuscript. All authors contributed to the article and approved the submitted version.

## Funding

This research was supported by funds from Craig H. Neilsen Foundation grant #351718 and NINDS grant #1R21NS116665-01 to SG.
