# Supplementary material for: Pharmacogenetic inhibition of TrkB signaling in adult mice attenuates mechanical hypersensitivity and improves locomotor function after spinal cord injury
Source: Front Cell Neurosci. 2022 Sep 26;16:987236. doi: 10.3389/fncel.2022.987236 (PMC9548551; doi:10.3389/fncel.2022.987236)
Supplement: Supplementary file 1 [file Presentation_1.pdf]

TITLE: Pharmacogenetic inhibition of TrkB signaling in adult mice attenuates mechanical hypersensitivity and improves locomotor function after spinal cord injury

Authors: Karmarcha K. Martin[†], Donald J. Noble[†], Shangrila Parvin, Kyeongran Jang and Sandra M. Garraway
Department of Cell Biology, Emory University School of Medicine, Atlanta, GA.
[†], Co-first authors.

Effect of TrkB inhibition in DRG neurons

Dissociation of DRG neurons

Electrophysiological and pharmacological studies were undertaken in dorsal root ganglion (DRG) neurons to assess the effect of 1NMP-induced TrkB inhibition of agonist-evoked inward current. Mice were treated with 1NMP in drinking water (as described above) for 5 days. Veh-treated (n=4) and 1NMP-treated (n=4) animals were deeply anesthetized and euthanized with isoflurane for DRG extraction. Dissociation and culture of DRG neurons was performed according to English et al. 2020. Briefly, DRGs were extracted from thoracic 4 – lumbar 2 spinal levels and placed in cold Hanks' Balanced Salt Solution (HBSS; Corning). The DRGs were then enzymatically digested in HBSS containing dispase and collagenase. Cells were dissociated by trituration through a set of fire-polished glass pipettes in neurobasal medium-A (NB-A; ThermoFisher) with 2% B-27 (ThermoFisher), 1% penicillin/streptomycin (Lonza Biowhittaker), and 1% Glutamax (ThermoFisher). Dissociated cells were then resuspended and seeded at low density on coverslips coated with laminin and poly-L-lysine. Plates were kept in a 37°C incubator with 5% CO_2 for at least 24 h before electrophysiological recording.

Whole cell recording from dissociated DRG neurons

Using standard whole-cell patch clamp procedures, recordings were made from small DRG neurons (soma diameter: 15 - 30 μm) using a MultiClamp 700B amplifier (Molecular Devices, Sunnyvale, CA). Patch electrodes were filled with solution containing (in mM) 140 K^+-gluconate, 11 EGTA, 10 HEPES, 1 $CaCl_2$, 4 Mg-ATP, and 1 Na-GTP (pH 7.4 adjusted with KOH, 320 mOsM). Artificial cerebrospinal fluid (aCSF) containing (in mM) 140 NaCl, 3 KCl, 2 $MgCl_2$, 1.8 $CaCl_2$, 10 glucose, 10 HEPES (pH 7.3 adjusted with KOH, 300 mOsM) was oxygenated with 95% O_2 – 5% CO_2 and continuously delivered to the recording chamber at room temperature. Signals were filtered at 1kHz and digitized at 10 kHz (Digidata 1440 A; Molecular Devices). Following determination of resting membrane potential and other electrophysiological properties, cells were held at -60 mV in voltage-clamp mode for pharmacological investigation described below.

Drug application

Contributions of BDNF and its receptor TrkB to hyperexcitability of dissociated DRG neurons were investigated by applying 7,8-dihydroxyflavone (7,8-DHF; Tocris Bioscience) to the aCSF bath at a concentration of 100 μM. 7,8-DHF has been identified to bind to the extracellular binding domain of TrkB, promote receptor autophosphorylation, and activate downstream signaling cascades that lead to physiological functions induced by binding of BDNF (Liu et al. 2016).

Results

Recordings were performed in 43 cells [Veh-treated (n=22) and 1NMP-treated (n=21)]. As shown in Supplemental Figure (S) 1A, 7, 8-DHF induced an inward current (267 ± 49 pA) in Veh-treated TrkBF616 mice. However, in mice treated with 1NMP for 5 days, there was a significant reduction in the amplitude of the induced current (47 ± 16) (p = .0001, *t test)*. Figure S1B shows examples of inward current traces during 7,8-DHF administration in neurons obtained from Vehicle and 1NMP treated mice.

Conclusion:

These results demonstrate that 1NMP treatment for 5 days effectively blocks TrkB agonist-induced responses, confirming that 1NMP treatment inhibits TrkB signaling in TrkBF616 mice.

Figure S1

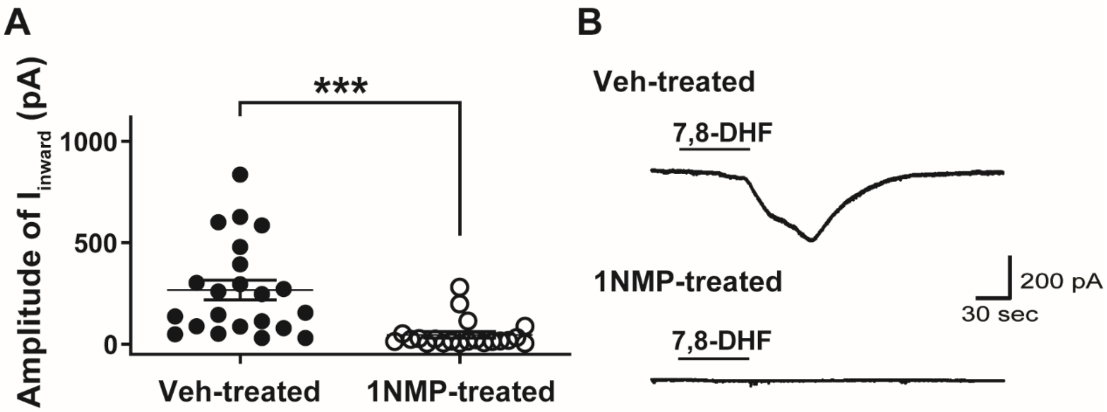

References

English AW, Liu X, Mistretta OC, Ward PJ, Ye K. Asparagine Endopeptidase (δ Secretase), an Enzyme Implicated in Alzheimer's Disease Pathology, Is an Inhibitor of Axon Regeneration in Peripheral Nerves. eNeuro. 2021 Jan 15;8(1):ENEURO.0155-20.2020. doi: 10.1523/ENEURO.0155-20.2020. PMID: 33323399; PMCID: PMC7814480.

Liu C, Chan CB, Ye K. 7,8-dihydroxyflavone, a small molecular TrkB agonist, is useful for treating various BDNF-implicated human disorders. Transl Neurodegener. 2016 Jan 6;5:2. doi: 10.1186/s40035-015-0048-7. PMID: 26740873; PMCID: PMC4702337.
